# The Cancer Research Fund

**Published:** 1909-07-24

**Authors:** 


### The Hospital

A Journal of

The Medical Sciences and Hospital Administration.

New Series. No. 126, Vol. V. [No. 1198, Vol. XLVI.] SATURDAY, JULY 24, 1909.

### The Cancer Research Fund.

This year the committee of the Imperial Cancer Research Fund do not intend to publish any scientific report, as the work which has been in progress is mainly a further development of the investigations summarised in the Third Scientific Report published last October, and is not yet sufficiently complete for paper and print. In these circumstances the medical world, and the large numbers of the laity who take an interest in the war against malignant disease, will study with extra attention the speech of Sir William Church in moving the adoption of the report at the annual meeting of the general committee, held under the presidency of His Royal Highness the Prince of Wales at Marlborough House on July 9. It would seem that the destructive work of the Fund is still not fully accomplished—the clearing away of the many myths and fallacies which have collected round the subject of cancer. Until this is thoroughly effected it would be as idle to count upon any great advance of constructive therapeutics as to attempt to build an enduring edifice on the debris of a former ruin. This is not to say that no progress will yet be made, but only that no absolute certainty of it can be predicted; it is quite possible that a happy chance might at any time provide some notable improvement in our methods for dealing with the scourge, but the workers under the Fund Committee are quite rightly averse to any reliance upon chance. Probably the most important of the researches now going on are those in connection with the increased proclivity of mice to cancer through inheritance. The figures available are as yet insufficient, Sir William Church says, for any definite conclusion; but the opinion seems to be gaining ground that heredity is a much less important factor in determining the incidence of malignant disease than has generally been supposed, if indeed it has any particular influence at all. In the nature of things breeding experiments take time, and, although the investigators have the good fortune to find in the mouse a mammal which is especially prone to carcinoma, is prolific, short-lived, tame, and easily handled and fed, yet even then it is obvious that some years must elapse before problems affecting the ætiology of disease in remote generations from a cancer-infected ancestry can be solved. Another point which has engaged the attention of the workers is the connection between implanted carcinoma and the development of sarcoma in the tissues of the host. A strain of carcinoma has been discovered which produces, when successfully inoculated, sarcomatous reaction in the tissues of the host almost constantly. What the precise significance of this peculiar fact may be is not obvious, but it at least shows how extraordinarily complex is the labyrinth in which the Cancer Research Fund are groping for clues.

Another important aspect of the case to which attention is being directed is the production of artificial immunity to cancer infection. Here a curious fact emerges. Whereas in diseases of definite origin the inoculation of killed cultures of the specific organism suffices to produce, in appropriate dosage, a high degree of artificial immunity, the same does not hold good of cancer. Artificial immunity, when it can be produced, is brought about only by the use of living cells (cancerous or not); and it is also noteworthy that whether physical or chemical means have been employed to destroy the vitality of cells which, when living, can confer immunity to cancer, the result is the same. Moreover when radium has been used in like manner, the immunising power of the cells is completely lost even though no microscopic structural change in the cells can be detected. Since this is true of both cancerous and non-cancerous protective cells, it is arguable that radium has no selective action on cancerous tissue. With respect to the treatment of malignant disease by trypsin and other ferments the report and Sir William Church are silent; but it would seem that facilities were afforded for independent investigation into this much-advertised and strongly criticised method of treatment with results completely fatal to the contentions of those who have advocated it. The history of the trypsin "boom" is highly instructive, for it is merely typical of too many pseudo-scientific efforts to treat disease. About five years ago the conception that the pancreatic digestive ferment might possibly have some direct local action upon carcinomatous cells originated in the mind of a certain gentleman who gave publicity to his idea. The method was tried upon a series of inoperable cases, and was believed to have done good. Seeing the possibilities of financial exploitation certain less reputable self-styled specialists adopted the treatment, which soon received considerable advertisement in the lay Press. Every unprejudiced observer who followed the directions given for the application of the remedy soon arrived at the conclusion that it was not merely valueless but actually harmful; but, notwithstanding this, for a time the public flocked to those who were more content to fill their own pockets than to study carefully the real results of the treatment they carried out. The published statements of those who felt they could not recommend the trypsin treatment were discounted by assertions that some essential detail of the technique had been rejected; and new combinations of ferments, each in turn absolutely infallible, superseded one another as the perfected form of the remedy. Confused by their inability to distinguish the caution of the true scientist from the blindness of unprogressive prejudice, the public could hardly be blamed for grasping at the proffered straws, any more than they can be for the credulity displayed in regard to many of the tuberculosis "cures" which have been boldly but dishonestly advocated by members of the medical profession in recent years. But if they will only take to heart the lesson of the trypsin incident and realise that leaps in the dark and brilliant guesses are essentially not the true way of scientific advance, but that the patient methods of the Cancer Research Committee is that which gives a real promise of solid progress in the future, then possibly out of evil may come good. For in that case it is unthinkable that the handicap of straitened circumstances shall be allowed to hinder a work which is neither for an age, but for all time; nor for a race, but for all the world.

